# Probiotic *Bifidobacterium longum* alters gut luminal metabolism through modification of the gut microbial community

**DOI:** 10.1038/srep13548

**Published:** 2015-08-28

**Authors:** Hirosuke Sugahara, Toshitaka Odamaki, Shinji Fukuda, Tamotsu Kato, Jin-zhong Xiao, Fumiaki Abe, Jun Kikuchi, Hiroshi Ohno

**Affiliations:** 1 Food Science and Technology Institute, Morinaga Milk Industry Co., Ltd., Zama, Kanagawa, Japan; 2 RIKEN Center for Integrative Medical Sciences, Yokohama, Kanagawa, Japan; 3 Graduate School of Medical Life Science, Yokohama City University, Yokohama, Kanagawa, Japan; 4 Institute for Advanced Biosciences, Keio University, Tsuruoka, Yamagata, Japan; 5 RIKEN Center for Sustainable Resource Science, Yokohama, Kanagawa, Japan

## Abstract

Probiotics are well known as health-promoting agents that modulate intestinal microbiota. However, the molecular mechanisms underlying this effect remain unclear. Using gnotobiotic mice harboring 15 strains of predominant human gut-derived microbiota (HGM), we investigated the effects of *Bifidobacterium longum* BB536 (BB536-HGM) supplementation on the gut luminal metabolism. Nuclear magnetic resonance (NMR)-based metabolomics showed significantly increased fecal levels of pimelate, a precursor of biotin, and butyrate in the BB536-HGM group. In addition, the bioassay revealed significantly elevated fecal levels of biotin in the BB536-HGM group. Metatranscriptomic analysis of fecal microbiota followed by an *in vitro* bioassay indicated that the elevated biotin level was due to an alteration in metabolism related to biotin synthesis by *Bacteroides caccae* in this mouse model. Furthermore, the proportion of *Eubacterium rectale*, a butyrate producer, was significantly higher in the BB536-HGM group than in the group without *B. longum* BB536 supplementation. Our findings help to elucidate the molecular basis underlying the effect of *B. longum* BB536 on the gut luminal metabolism through its interactions with the microbial community.

Probiotics are defined as live microorganisms that confer a health benefit to the host when administered in adequate amounts (FAO/WHO 2002), and they have been demonstrated to improve intestinal conditions toward the amelioration of irritable bowel syndrome and inflammatory bowel disease to prevent infectious diarrhea and inhibit severe necrotizing enterocolitis^1^,^2^,^3^. The probiotic effect on the modulation of intestinal environments is considered the principal and fundamental effect of probiotics and is acknowledged as the basis of other probiotic benefits. However, the gut microbial metabolism underlying the modulation of intestinal environments is far from understood, likely due to the complex interactions within the gut microbial community.

In recent years, “omics” techniques, including metabolomics^4^,^5^ genomics^6^, and transcriptomics^7^, have been developed to understand the “whole picture” of biological reactions in cells, organs, and organisms. Furthermore, germ-free and gnotobiotic animal models have been applied as a powerful tool for understanding the metabolism of the gut luminal environment^8^. The combination of these “omics” approaches and gnotobiotic mouse models can be used to evaluate microbial crosstalk in the complex gut microbial community. The use of these techniques demonstrated that the preventive effect of probiotic strains of *Bifidobacterium longum* against enterohemorrhagic *Escherichia coli* infection is based on the high carbohydrate metabolism of bifidobacterial strains followed by the production of acetate, a short-chain fatty acid, which upregulates a barrier function of the host gut epithelium^9^. Animal models with human gut microbiota (HGM) have been used to demonstrate the beneficial effect of probiotics. McNulty *et al*. used a gnotobiotic mouse model harboring 15 predominant genome-sequenced species of HGM and revealed that gut microbial gene expression related to plant polysaccharide metabolism was significantly upregulated by supplementation with fermented milk strains containing bifidobacteria^10^. Similar changes induced by supplementation with fermented milk were also observed in a clinical trial^10^. These gnotobiotic mouse studies, combined with omics approaches, have the potential to provide a translational research pipeline for the characterization of the crosstalk between probiotics and the human gut microbial community.

The *Bifidobacterium longum* BB536 (denoted *B. longum* BB536) strain is a probiotic strain that has been reported to have various physiological effects, such as anti-allergy effects^11^, reductions in harmful bacteria^12^,^13^, and improvements in the intestinal environment, defecation frequency and stool characteristics^14^,^15^. However, in human studies, the molecular mechanisms of these effects remain obscure. Due to the complex interactions within the gut microbial community, the effects of *B. longum* BB536 on the types of gut luminal metabolism affected are not known.

In this study, we evaluated the effect of *B. longum* BB536 on the modulation of gut environments in gnotobiotic mice harboring 15 species of HGM using multifaceted approaches, including metabolome, 16S rRNA gene metagenome and metatranscriptome analyses. We found that supplementation of the *B. longum* BB536 strain altered gut luminal biotin and butyrate metabolism through a modification of the gut microbial community.

## Results

### NMR-based metabolomics on water-soluble fecal metabolites of the HGM and BB536-HGM groups

**Figure 1:**
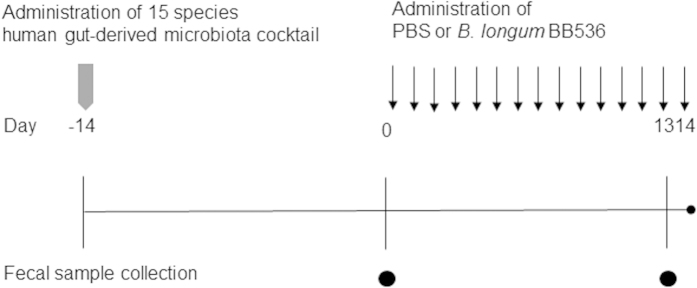
Experimental design of the mouse study. Germ-free mice were inoculated with a single gavage of 15 species of a predominant human gut-derived microbiota cocktail at day -14 and were administered PBS containing *Bifidobacterium longum* BB536 or nothing (n = 6) every day for 14 days. Fecal samples were collected on days 0 and 13.

**Figure 2:**
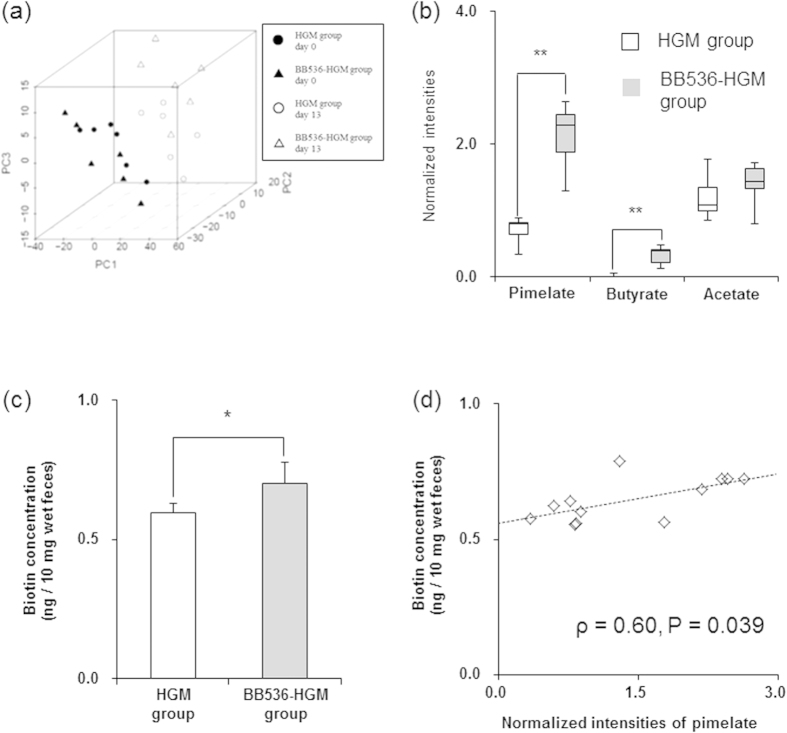
Water-soluble metabolic profiling in fecal samples of the human gut-derived microbiota (HGM) mice. (**a**) Principal component analysis (PCA) of fecal 1D ^1^H-NMR spectroscopy profiles. The contributions of PC1, PC2 and PC3 were 63.7%, 20.1% and 6.3%, respectively. (**b**) Fecal pimelate, butyrate and acetate levels at day 13. All compounds were calculated and identified using ^1^H-^13^C HSQC 2D-NMR measurements (n = 6). Boxes denote the interquartile range between the first and third quartiles, and the line within denotes the median. P-values were calculated using Mann–Whitney U test. *P < 0.05; **P < 0.01. (**c**) Biotin concentrations in fecal samples at day 13. Data are shown as the mean ± SD (n = 6). P-values were calculated using Student’s t-test. *P < 0.05. (**d**) Correlation analysis based on Spearman’s rank correlation coefficient between fecal pimelate and biotin levels. Fecal pimelate and biotin levels are shown as open symbols, and the line indicates a simple regression model.

Fecal samples collected at days 0 and 13 were analyzed using NMR-based metabolomics to evaluate the effect of *B. longum* BB536 supplementation on the metabolites in the gut environments in HGM mice (Fig. 1). Principal component analysis (PCA) of ^1^H-NMR data of fecal extracts from the HGM and BB536-HGM groups revealed different clusters on day 13 (Fig. 2a). ^1^H-^13^C heteronuclear single-quantum coherence (HSQC)-based two-dimensional NMR (2D-NMR) analysis of the fecal samples indicated that the normalized intensities of pimelate (P = 0.002), a precursor of biotin^16^, and butyrate (P = 0.002) were significantly higher in the BB536-HGM group than in the HGM group (Fig. 2b). The other metabolites detected by 2D-NMR metabolomics, including acetate (Fig. 2b), were comparable between these two groups (data not shown).

Pimelate is a substrate in microbial biotin synthesis pathways^16^. Because the biotin concentrations in examined feces were under the detection limit of the metabolomic approach, we analyzed the biotin concentrations with a highly sensitive assay using *Lactobacillus plantarum* ATCC 8014. This highly sensitive assay confirmed the higher biotin concentration in the feces of the BB536-HGM group compared to the HGM group (Fig. 2c). The normalized intensity of pimelate detected by 2D-NMR metabolomics was consistently and significantly correlated with the concentration of biotin as determined using the highly sensitive assay (ρ = 0.60, P = 0.039) (Fig. 2d).

### Comparison of fecal metabolic profiles between germ-free (GF) and *B. longum* BB536 mono-associated (BB536-MA) mice

**Figure 3:**
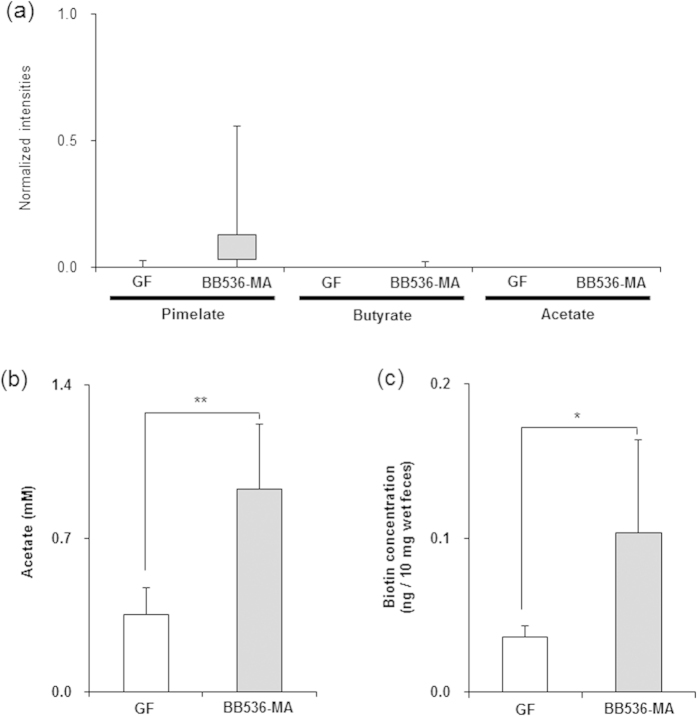
Metabolite levels in germ-free and *Bifidobacterium longum* mono-associated mice. (**a**) Fecal pimelate, butyrate and acetate levels measured by 2D-NMR. The levels were based on the normalized intensities calculated from ^1^H-^13^C HSQC 2D-NMR measurements (n = 5). Boxes denote the interquartile range between the first and third quartiles, and lines within the boxes denote the median. P-values were calculated using the Mann–Whitney U test. (**b**) Acetate concentration in fecal samples measured by enzyme method. Data are shown as the mean ± SD (n = 5). P-values were calculated using Student’s t-test. **P < 0.01. (**c**) Biotin concentration in fecal samples after treatment. Data are shown as the mean ± SD (n = 5). P-values were calculated using Student’s t-test. *P < 0.05.

Germ-free mice that were mono-associated with *B. longum* BB536 were employed to determine whether changes in the fecal metabolites in the BB536-HGM group, including increases of pimelate, biotin, and butyrate, were induced by *B. longum* BB536 or by interactions with the HGM strains. The fecal metabolites of GF mice and BB536-MA mice were observed using 2D-NMR metabolomics; however, the signal intensities of pimelate, butyrate and acetate by 2D-NMR were under the detection limits (Fig. 3a). Therefore, ^1^H-NMR analysis, which has a higher sensitivity than 2D-NMR, was performed to evaluate the differences between the two groups. The predicted signal intensity of acetate was significantly higher in BB536-MA mice than in GF mice (P = 0.008), whereas significant differences were not observed in the predicted signal intensities of pimelate and butyrate between the two groups (Supplementary Figure S1). The difference in the predicted signal intensity of acetate was confirmed by an enzymatic analysis (P=0.004) (Fig. 3b). In addition, a significantly higher level of fecal biotin was detected in BB536-MA mice than in GF mice (P = 0.037) (Fig. 3c) when assayed using the highly sensitive assay.

### Effect of *B. longum* BB536 supplementation on gut microbiota in gnotobiotic mice associated with 15 species of predominant human gut-derived microbiota (HGM mice)

The fecal microbiota profiles of HGM mice administered PBS (HGM group) or *B. longum* BB536 (BB536-HGM group) based on the V3–V4 region of bacterial 16S rRNA genes were analyzed (Table 1). The proportion of *Bacteroides vulgatus* was significantly lower in the BB536-HGM group than in the HGM group on day 13 of the administration period (P = 0.041), whereas the proportions of *Eubacterium rectale* (denoted *E. rectale*) (P = 0.015) and *B. longum* (P = 0.002) were significantly higher in the BB536-HGM group.

### Identification of the genes related to the metabolic changes altered by BB536 supplementation in gut microbiota of HGM mice

**Figure 4:**
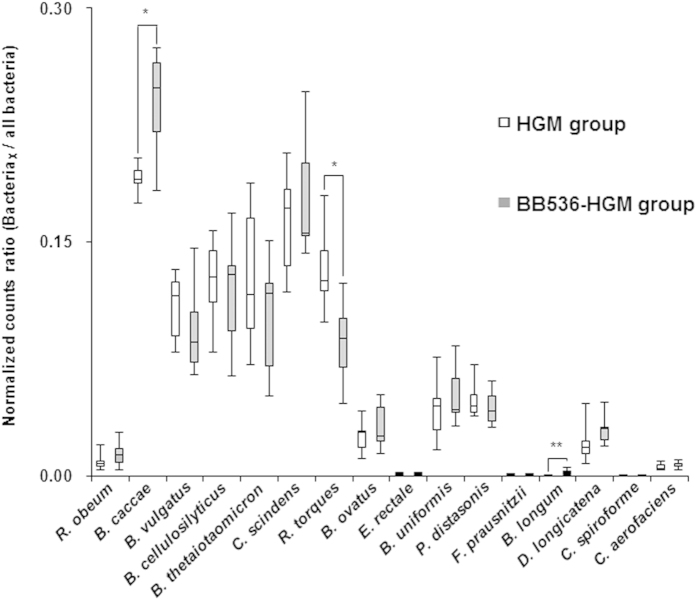
Distribution of bacterial transcripts in fecal samples of the human gut-derived microbiota (HGM) mice. Boxes denote the interquartile range between the first and third quartiles, and the line within denotes the median. P-values were calculated using the Mann–Whitney U test. *P < 0.05; **P < 0.01.

Metatranscriptome analysis indicated that the gene transcript ratios of *Bacteroides caccae* (denoted *B. caccae*) (P = 0.041) and *B. longum* (P = 0.002) were significantly higher and that of *Ruminococcus torques* (P =0.015) was significantly lower in the BB536-HGM group than in the HGM group, suggesting that these changes induced by *B. longum* BB536 might be responsible for the gut luminal metabolic changes, such as biotin and butyrate production, in the BB536-HGM group (Fig. 4).

**Figure 5:**
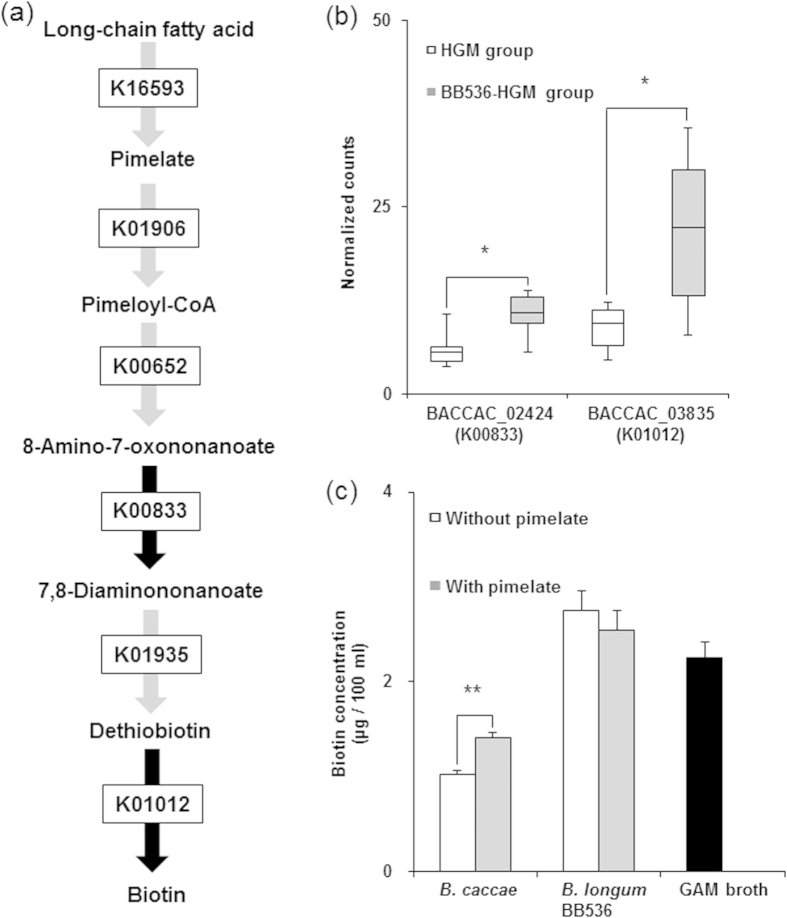
Predicted contribution to biotin production in gut microbiota. (**a**) Components of pathways from pimelate to biotin. The black arrow indicates the step with a significant difference between the two groups. (**b**) Gene expression with a significant difference between the two groups. RPKM-normalized reads are shown as boxes that denote the interquartile range between the first and third quartiles, and the line within denotes the median (n = 6). P-values were calculated using the Mann–Whitney U test. *P < 0.05. (**c**) *In vitro* assay of biotin synthesis. Each strain was cultivated for 16 h in GAM broth with or without pimelate (10 mg/ml). Data are shown as the mean ± SD (n = 3). P-values were calculated using Student’s t-test. **P < 0.01.

We then addressed the metabolic pathways of each microbial species using blastx program in the Basic Local Alignment Search Tool (BLAST) or K numbers in the Kyoto Encyclopedia of Genes and Genomes (KEGG) database^17^ to identify bacterial species that contribute to pimelate, biotin and butyrate production. First, the blastX program identified genes that were homologous with cytochrome P450_BioI_, an enzyme with a reported relationship with pimelate synthesis^18^,^19^,^20^,^21^, in *Faecalibacterium prausnitzii*, *Dorea longicatena* and *Bacteroides uniformis* (FAEPRAA2165_02218, DORLON_01762, BACUNI_02910); however, significant differences were not observed between the HGM and BB536-HGM group (Supplementary Table S1). In addition, two gene transcripts of *B. caccae* (*BACCAC*_02424: P = 0.041; *BACCAC*_03835: P = 0.041) were significantly increased in the BB536-HGM group compared with the HGM group (Fig. 5a–b). These genes are involved in the biotin synthesis pathway, which proceeds through pimelate metabolism. The expression levels of other genes in the biotin synthesis pathway were comparable between the HGM and BB536-HGM groups (Supplementary Figure S2). *In vitro* cultivation of *B. caccae* with or without pimelate showed consistent and significant accumulations of biotin in the pimelate supplementation group (Fig. 5c) (P = 4.4 × 10–4). Microbial gene expression levels in the butyrate production pathway were comparable between the HGM and BB536-HGM groups (Supplementary Figure S3).

## Discussion

The human gut harbors a wide variety of commensal microbiota that play fundamental roles in the well-being of their host^22^. Many studies have discussed the microbial fermentation that transforms indigestible dietary fibers, such as glycans, into short-chain fatty acids that serve as nutrients for host colonocytes and other gut epithelial cells^23^. Furthermore, gut microbiota synthesize most types of B vitamins, such as biotin, folates, cobalamin, nicotinic acid, pantothenic acid, pyridoxine, riboflavin and thiamine^24^. In contrast to dietary vitamins, which are mostly absorbed in the proximal part of the small intestine, the uptake of microbial fermentation-derived vitamins occurs predominantly in the colon, which contributes to the maintenance of systemic vitamin levels^25^. However, the molecular mechanisms underlying the gut microbiota synthesis of B vitamins in the gut lumen have not been well elucidated.

The multifaceted omics approaches used in this study showed that supplementation with *B. longum* BB536 altered the gut microbial community, gene expression, and fecal metabolite concentrations, such as butyrate and pimelate, in HGM mice. Because the fecal concentrations of both butyrate and pimelate did not increase in BB536-MA mice, *B. longum* BB536 is thus speculated to increase gut luminal butyrate and pimelate concentrations through microbial crosstalk with HGM. *B*. *longum* BB536 was found to possess some ability to produce biotin in the gut luminal environment, although the metatranscriptome analysis and *in vitro* assay also suggested that pimelate contributed to the increments of fecal biotin levels.

Arumugam *et al*. reported that biotin biosynthesis genes (8-amino-7-oxononanoate synthase: K00652; adenosylmethionine-8-amino-7-oxononanoate aminotransferase: K00833; dethiobiotin synthetase: K01935) contributed to the clustering of enterotype 1, which is enriched in the *Bacteroides* genus^26^. Our metatranscriptome analysis also revealed a higher expression of genes related to biotin biosynthesis^27^,^28^ for *B. caccae* in the BB536-HGM group compared to the HGM group. In addition, *in vitro* bioassays indicated that *B. caccae* metabolized pimelate into biotin. Taken together, these results indicate that the increase in biotin concentrations in the gut lumen of these HGM mice was induced by the co-existence of *B. longum* BB536 and *B. caccae*.

Few studies have examined the physiological effects of pimelate as well as the microbial metabolic pathways related to pimelate synthesis, although studies have suggested that pimelate might also be produced from long-chain fatty acids via the enzymatic reaction of cytochrome P450_BioI_^18^,^19^,^20^,^21^. We found the existence of genes that are homologous with cytochrome P450_BioI_; however, differences in the expression levels of these homologous genes were not observed after supplementation with *B. longum* BB536 (Supplementary Table S1). Therefore, further analysis is required to understand the molecular basis of the pimelate increases resulting from *B. longum* BB536 supplementation in HGM mice and the physiological function of pimelate produced in the colon.

Our study using HGM mice also found that supplementation with *B. longum* BB536 increased the fecal butyrate concentration. The 16S rRNA gene-based microbiome analysis revealed that the proportion of *E. rectale* in HGM mice decreased significantly compared to baseline in both groups (HGM group: P = 0.046; BB536-HGM group: P = 0.028). The reason for this result is still unclear, but it may be due to a failure to reach a steady state during the 2 weeks of acclimation or oral administration in this study. Nevertheless, the proportion of *E. rectale* was significantly higher in the group supplemented with *B. longum* BB536 (P = 0.015). *E. rectale* produces butyrate from acetate^29^, one of the main fermentation products from carbohydrates produced by bifidobacteria^30^. In fact, Falony *et al*. showed that acetate produced by *B. longum* BB536 promoted growth of butyrate-producing bacteria and the *in vitro* production of butyrate^31^. Therefore, the increase in the fecal butyrate level caused by *B. longum* BB536 supplementation in HGM mice might be at least partially due to the metabolism of acetate into butyrate by butyrate-producing bacteria.

In this HGM mouse model, *B. caccae* and *E. rectale* were supposed to be involved in the production of biotin and butyrate, respectively. However, further studies are needed to clarify the contribution of these bacterial species using an HGM mouse model lacking these bacteria or other models. In addition, knowledge is needed to connect these results to humans. Previous studies have shown the intense efficacy of *B. longum* BB536 in improving the intestinal environment, such as reducing harmful bacteria and improving defecation frequency and stool characteristics^12^,^13^,^14^,^15^,^32^. However, there have been no reports on the changes in fecal butyrate and/or biotin/pimelate. It has been reported that prebiotic fructooligosaccharide supplementation in healthy human volunteers for one week increased fecal butyrate concentrations and Bifidobacteriaceae proportions^33^. Veiga *et al*. also showed that supplementation of fermented milk product containing a *Bifidobacterium* strain induced increases in butyrate-producing bacteria and butyrate concentrations in the gut luminal environment^34^.

It is known that butyrate induces the differentiation of colonic Treg cells, which contribute to anti-inflammatory effects on epithelial cells^35^. Biotin is an essential nutrient for mammals, including humans. It enhances glucose-stimulated insulin secretion in isolated perfused pancreas^36^ and influences lymphocyte maturation^37^. Further studies are necessary to reveal the effects of *B. longum* BB536 supplementation on fecal butyrate and pimelate/biotin levels in humans and to understand the physiological effects to the host mediated by the production of these metabolites.

**Figure 6:**
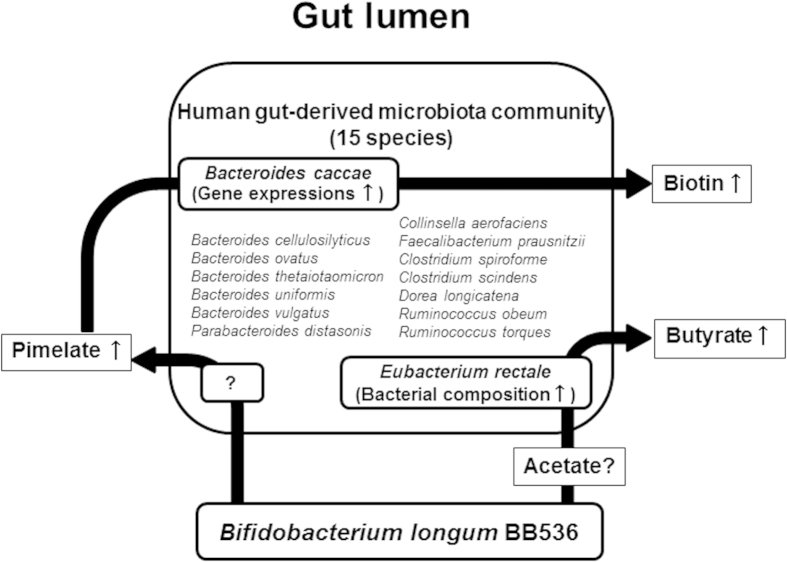
Proposed effect of *Bifidobacterium longum* BB536 on the gut luminal metabolism mediated by interaction with the human gut-derived microbiota (HGM) community.

In conclusion, using an HGM mouse model, we demonstrated that *B. longum* BB536 supplementation increased the amounts of fecal pimelate, biotin and butyrate, likely through microbial crosstalk among *B. longum* BB536 and human gut-derived microbiota (Fig. 6). To our knowledge, this report is the first observance of the effects of probiotics on biotin metabolism in the gut luminal environment. Our findings provide new insights into the beneficial effects of this probiotic strain on gut luminal metabolism through the modulation of the gut microbial community.

## Methods

### Bacterial strains and culture conditions

Human gut-derived microbial species were selected based on a previous study^10^ and were obtained from public culture collections (Supplementary Table S2). Pre-cultures of *Bacteroides cellulosilyticus* JCM15632 and *Bacteroides uniformis* JCM5828 were prepared by culturing at 37 °C for 16 h in Gifu Anaerobic Medium broth (GAM broth, NissuiSeiyaku Co. Ltd., Tokyo, Japan). Other strains were pre-cultivated in modified EG medium (Supplementary Table S3) at 37 °C for 16 h. All human gut-derived microbial species were cultivated in modified EG medium at 37 °C for 24 h after pre-cultivation. The McFarland nephelometric method (DEN-1B, Wakenbtech, Kyoto, Japan) was used to estimate cell numbers of human gut-derived microbial species. *B. longum* BB536 was obtained from the Morinaga culture collection (Morinaga Milk Industry Co. Ltd., Kanagawa, Japan), and the cells were anaerobically cultivated in LACTOBACILLI MRS broth (BD Difco, Sparks, MD) containing 0.05% L-cysteine · HCl.

### Animal experiments

GF BALB/c mice (8 weeks, female) were obtained from the Sankyo Labo Service Corporation (Tokyo, Japan) and inoculated with a single gavage of modified EG medium containing 15 species of human gut-derived microbiota (approximately 3 × 10^6^ cells of each strain). Two weeks later, phosphate-buffered saline (PBS) or *B. longum* BB536 suspension in PBS (1 × 10^9^ CFU, BB536-HGM) were administered to HGM mice daily for 14 days. Fecal samples were collected on days 0 and 13 (Fig. 1). In *B. longum* BB536-monoassociated mice studies, GF BALB/c mice (33–39 weeks, male and female) were orally inoculated with PBS or a *B. longum* BB536 suspension in PBS (1 × 10^7^ CFU), and fecal samples were collected 13 days after inoculation. All mice were fed γ-ray-sterilized AIN-93M (Funabashi farm, Chiba, Japan). All animal experiments were approved by the Animal Research Committees of Yokohama City University and were performed in accordance with the Guide for the Care and Use of Laboratory Animals of Yokohama City University.

### H-NMR and ^1^H-^13^C HSQC 2D-NMR measurements

All NMR-based analyses were performed as described previously, with some modifications^9^,^35^. Briefly, water-soluble fecal metabolites were extracted at 65 °C for 15 min by gentle shaking with 100 mM potassium phosphate buffer containing 90% deuterium oxide and 1 mM sodium 2,2-dimethyl-2-silapentane-5-sulphonate as the chemical-shift reference compound (δ = 0.0 ppm) and were analyzed using ^1^H-NMR and 2D-NMR. All NMR measurements were conducted using a DRX-700 spectrometer equipped with a cryogenically cooled probe. NMR spectra were processed as described previously, with some modifications^4^,^5^,^38^,^39^. Briefly, ^1^H-NMR data were reduced by subdividing the spectra into sequential 0.04 ppm designated regions between ^1^H chemical shifts of 0–9.5 ppm, and water resonance was excluded using Automics (beta version 0.99 .1220)^40^. Each region was integrated and normalized to the total of all resonance integral regions. The PCA was run on R software using the scatterplot3d package. Each coordinate on the score plot represents an individual sample. Each ^1^H-^13^C HSQC spectrum was recorded, and a region of interest (ROI) was created for each peak using rNMR (ver. 1.1.7)^41^. Thereafter, the area intensity in each ROI was normalized with internal standards (sodium 2,2-dimethyl-2-silapentane-5-sulphonate) and then used for statistical analysis. Metabolite assignments were performed using a previously reported database^42^,^43^ with ^1^H (±0.05) and ^13^C (±0.53) tolerances.

### Biotin measurement

Fecal material was homogenized in PBS and centrifuged. The supernatants were filtered. Fecal concentrations of free biotin were determined with a highly sensitive assay using *Lactobacillus plantarum* ATCC 8014 as previously described^44^.

### Acetate measurement by enzymatic analysis

Acetate concentrations in the fecal samples were determined by enzymatic analysis using an F-kit (Roche Diagnostics, Mannheim, Germany) as previously described^45^.

### Comparative fecal microbiome analysis

DNA was extracted from fecal samples using the bead-beating method^46^, and 16S rRNA gene sequencing was performed as described previously with some modifications^47^. The V3–V4 region of bacterial 16S rRNA genes were amplified using PCR with a TaKaRa Ex Taq HS kit (TaKaRa Bio, Shiga, Japan) and a primer set of Tru357F (5′-CGCTCTTCCGATCTCTG TACGGRAGGCAGCAG-3′) and Tru806R (5′-CGCTCTTCCGATCTGAC GGACTACHVGGGTWTCTAAT-3′). DNA was amplified according to the following program: preheating at 94 °C for 3 min; 25 cycles of denaturation at 94 °C for 30 s, annealing at 50 °C for 30 s and extension at 72 °C for 30 s; and a final terminal extension at 72 °C for 10 min. The 16S rRNA gene amplicons were then amplified with a 2nd primer set, adapted for the Illumina MiSeq (Illumina, San Diego, CA), as shown in Supplementary Tables S4–5. DNA was amplified according to the same program as indicated above, except for cycle number 15. The 2nd amplified PCR products were purified using a QIAquick PCR Purification Kit (Qiagen) according to the manufacturer’s protocol. Purified products were quantified using a Quant-iT PicoGreen dsDNA Assay kit (Life Technologies, Carlsbad, CA). Subsequently, equal amounts of amplicons from different samples were pooled and removed primer-dimer by gel-extraction with the QIAquick PCR Purification Kit. The pooled libraries were sequenced using an Illumina MiSeq instrument with a MiSeq v3 Reagent kit (Illumina, San Diego, CA).

Data analysis was performed as described previously with some modifications^6^. The Illumina paired-end reads that passed the quality filters were combined using the fastq-join script in ea-utils (ver. 1.1.2–537)^48^. Potential chimeric sequences were removed by reference-based chimera checking in USEARCH (ver. 5.2.32)^49^ and the gold database (http://drive5.com/otupipe/gold.tz). The non-chimeric sequences were analyzed in the QIIME software package, version 1.7.0^50^,^51^, using closed-reference operational taxonomic unit (OTU) picking against 16S rRNA genes of the 15 species of predominant human gut-derived microbiota and *B. longum* BB536.

### Bacterial RNA extraction

Fresh fecal samples were collected in a plastic tube containing RNAprotect Bacteria Reagent (Qiagen). The samples were centrifuged at 5000 g for 10 min, and the pellet was frozen immediately at −80 °C until use. Bacterial RNA extraction was performed as described previously with a few modifications^52^. Briefly, the pellet was suspended and homogenized in 200 μl Tris-EDTA buffer (10 mM Tris-HCl, 1 mM EDTA, pH 8) containing 30 mg/ml lysozyme (SIGMA-ALDRICH, Tokyo, Japan), 5000 U/ml mutanolysin (SIGMA-ALDRICH) and 20 mg/ml proteinase K (Qiagen). The samples were incubated with gentle shaking for 60 min at room temperature, and 700 μl of RLT buffer (Qiagen) was added to the lysates. Total RNA was purified using RNeasy Mini kit (Qiagen) with an on-column DNase treatment following the manufacturer’s protocol.

### Metatranscriptome analysis

RNA-seq was performed as described previously with some modifications^53^. Briefly, ribosomal RNA was removed from bacterial RNA using a Ribo-Zero Magnetic Kit for Bacteria (Epicentre, Madison, WI). The final Ribo-Zero RNA sample was subjected to a ScriptSeq RNA-Seq v2 Library preparation kit (Epicentre). The length of the fragmented cDNA was checked using Agilent 2100 Bioanalyzer electropherograms (Agilent Technologies, Santa Clara, CA). Then, the barcoded cDNA libraries were pooled together with equal concentrations in one pool and run in a MiSeq instrument using a MiSeq v2 Reagent kit.

RNA-seq data were analyzed as described previously with some modifications^10^. The obtained paired-end reads were separated by barcode (Supplementary Table S6), and each paired-end read was trimmed and mapped against protein-coding sequences (Supplementary Table S2) and normalized to reads/kb gene length/million mapped reads (RPKM)^54^ by CLC Genomics Workbench v6.5 (CLCbio Japan, Tokyo, Japan) with default parameters and an “include broken pairs” counting scheme. Fragment sizes were set at a minimum length of 50 bp and a maximum length of 2000 bp. RPKM-normalized reads were combined at individual bacteria or all bacteria to evaluate fluctuations in whole-gene expression, and combined data were compared as proportions of individual bacteria to all bacteria. Thereafter, RPKM-normalized reads were compared in individual bacterial genes with K numbers, which are described in an IMG database (version 3.5)^55^ or our database.

### Prediction of cytochrome P450_BioI_

The enzyme cytochrome P450_BioI_ was predicted from all of the protein coding sequences analyzed with the blastX program (stand-alone BLAST version 2.2.28+) with an E-value (<1.0 × 10^−5^). The amino acid sequence of cytochrome P450_BioI_ derived from *Bacillus subtilis* subsp. *subtilis* 168 (BSU30190 described in KEGG database) was used as a reference database.

### *In vitro* assay of biotin synthesis from pimelate by gut microbes

*B. caccae* and *B. longum* BB536 were anaerobically cultured in fresh GAM broth adjusted to pH 6.8 with or without 10 mg/ml pimelate at 37 °C for 16 h using AnaeroPack (Mitsubishi Gas Chemical, Tokyo, Japan). The samples were centrifuged at 10,000 g for 10 min, and the supernatants were filtered and used for the highly sensitive assay.

### Statistical analyses

Analyses were performed using SPSS version 14.0 statistical software (SPSS Inc., Chicago, IL). For all analyses, P-values of < 0.05 were considered statistically significant.

### Data deposition

DNA sequences of 16S rRNA gene metagenome and metatranscriptome data were deposited at DDBJ under accession numbers DRA002536 and DRA002537, respectively.

## Additional Information

**How to cite this article**: Sugahara, H. *et al*. Probiotic *Bifidobacterium longum* alters gut luminal metabolism through modification of the gut microbial community. *Sci. Rep*. **5**, 13548; doi: 10.1038/srep13548 (2015).

## Supplementary Material

Supplementary Information

## Figures and Tables

**Table 1:** Microbiota profiles in fecal samples of HGM mice.

Species	Median percent of total reads (interquartile range)							
	Day 0				Day 13			
	HGM group		BB536-HGM group		HGM group		BB536-HGM group	
*R. obeum*	24.06	(23.28–24.87)	27.36	(23.32–28.27)	23.08	(22–25.28)	23.68	(22.97–34.50)
*B. caccae*	20.85	(18.07–21.5)	20.89	(19.99–22.37)	19.82	(19.7–20.51)	22.13	(18.23–24.36)
*B. vulgatus*	12.77	(12.6–14.56)	12.24	(10.13–13.39)	17.14	(15.73–18.53)	13.66	(12.29–14.24)*
*B. cellulosilyticus*	13.95	(13.37–14.96)	13.63	(12.46–14.64)	14.13	(12.82–15.46)	12.80	(11.80–14.14)
*B. thetaiotaomicron*	10.63	(9.95–11.7)	10.71	(10.63–11.18)	14.11	(12.83–15.64)^#^	13.06	(9.89–14.17)
*C. scindens*	4.25	(3.68–4.97)	5.18	(3.84–7.35)	4.08	(3.67–4.98)	4.15	(3.66–4.94)
*R. torques*	3.03	(2.34–4.06)	2.05	(1.38–2.4)*	3.05	(2.54–3.54)	2.88	(2.61–3.13)^#^
*B. ovatus*	4.16	(4.03–5.07)	3.78	(3.50–4.28)	0.25	(0.18–0.30)^#^	0.15	(0.13–0.18)^#^
*E. rectale*	1.92	(1.39–2.18)	1.96	(1.87–2.52)	1.01	(0.84–1.03)^#^	1.17	(1.09–1.27)^#,^*
*B. uniformis*	1.69	(1.53–1.97)	1.79	(1.43–1.90)	1.32	(1.28–1.35)	1.31	(1.14–1.52)^#^
*P. distasonis*	0.54	(0.35–0.78)	0.40	(0.31–0.61)	0.50	(0.34–0.68)	0.61	(0.45–0.80)
*F. prausnitzii*	0.51	(0.50–0.56)	0.51	(0.38–0.53)	0.45	(0.40–0.52)	0.46	(0.44–0.51)
*B. longum*	0.06	(0.05–0.06)	0.07	(0.05–0.08)	0.06	(0.02–0.08)	0.63	(0.52–0.69)^#,^**
*D. longicatena*	0.02	(0–0.04)	0.02	(0.02–0.04)	0.04	(0.02–0.05)	0.05	(0.02–0.06)
*C. spiroforme*	0.01	(0–0.02)	0	(0–0.05)	0	(0–0)	0	(0–0)
*C. aerofaciens*	0	(0–0)	0	(0–0)	0	(0–0)	0	(0–0)

Intra- and inter-group differences were analyzed using the Wilcoxon matched-pair signed-rank test and the Mann–Whitney U test, respectively. ^#^P < 0.05 for significant intra-group differences from baseline (day 0). *P < 0.05; **P < 0.01 for significant inter-group differences compared to the HGM group.
