# Supplementary material for: Probiotic Bifidobacterium longum alters gut luminal metabolism through modification of the gut microbial community
Source: Sci Rep. 2015 Aug 28;5:13548. doi: 10.1038/srep13548 (PMC4552000; doi:10.1038/srep13548)
Supplement: Supplementary Information [file srep13548-s1.pdf]

-Supplementary Information-

Probiotic *Bifidobacterium longum* alters gut luminal metabolism through modification of the gut microbial community

Hirosuke Sugahara[1,2,3*], Toshitaka Odamaki[1], Shinji Fukuda[2,4], Tamotsu Kato[2,3], Jin-zhong Xiao[1], Fumiaki Abe[1], Jun Kikuchi[3,5], Hiroshi Ohno[2,3]

[1] Food Science and Technology Institute, Morinaga Milk Industry Co., Ltd., Zama, Kanagawa, Japan, [2] RIKEN Center for Integrative Medical Sciences, Yokohama, Kanagawa, Japan, [3] Graduate School of Medical Life Science, Yokohama City University, Yokohama, Kanagawa, Japan, [4] Institute for Advanced Biosciences, Keio University, Tsuruoka, Yamagata, Japan, [5] RIKEN Center for Sustainable Resource Science, Yokohama, Kanagawa, Japan

Inventory of Supplementary Information

1. Supplementary Methods

2. Supplementary Note

3. Supplementary Figures and Legends
Supplementary Figure S1
Supplementary Figure S2
Supplementary Figure S3
Supplementary Figure S4

4. Supplementary Table
Supplementary Table S1
Supplementary Table S2
Supplementary Table S3
Supplementary Table S4
Supplementary Table S5
Supplementary Table S6
Supplementary Table S7
Supplementary Table S8

1. Supplementary Methods

Evaluation of changes in biotin production

Each of the bacterial strains used in this study was cultured in fresh GAM broth at 37 °C for 16 h using AnaeroPack (Mitsubishi Gas Chemical, Tokyo, Japan). The samples were centrifuged at 10,000 g for 10 min, and the supernatants were filtered and used in a highly sensitive assay to measure the biotin concentrations. Changes in biotin concentration were calculated by subtracting the average internal biotin level in the GAM broth from the biotin level in each sample. The bacterial composition of the biotin producer or consumer in the fecal microbiota was calculated by summing each value of 16S rRNA gene-based data.

2. Supplementary Note

Evaluation of bacterial composition according to the bacterial properties during biotin metabolism

We evaluated the properties of each bacterial strain during biotin metabolism through *in vitro* assays. The results indicate that *Bacteroides ovatus*, *Bacteroides vulgatus*, *Ruminococcus obeum*, *Faecalibacterium prausnitzii* and *Bifidobacterium longum* BB536 (denoted *B. longum* BB536) were biotin producers, whereas the remaining strains were biotin consumers (Supplementary Figure S4 a). The composition of biotin-producing or biotin-consuming bacteria at day 13 was insignificant between the HGM and BB536-HGM group (Supplementary Figure S4 b).

These results suggest that the bacterial composition of the HGM community with regards to biotin producers or biotin consumers was not affected by supplementation with *B. longum* BB536 (Supplementary Figure S4 a-b). Compared with the increased levels of biotin observed in the B536-HGM group, our results showed that the proportions of *Bacteroides vulgatus* (biotin producer) were significantly lower in the BB536-HGM group than in the HGM group

(P=0.041), whereas the proportions of *Eubacterium rectale* (biotin consumer) were significantly higher in the BB536-HGM group than in the HGM group (Table1, Supplementary Figure S4 a) (P=0.015), which might produce decreased levels of fecal biotin. Therefore, the increments of fecal biotin levels by *B. longum* BB536 supplementation may be affected by other mechanisms in addition to the modulation of gut microbiota composition. We suggest that variations in the gene expression of *Bacteroides caccae* and metabolism of pimelate may act as mechanisms that increase the levels of fecal biotin induced by *B. longum* BB536 supplementation. Ifuku *et al*. reported that bacteria utilize acetate for biotin synthesis[1]. Therefore, acetate produced by bifidobacteria might contribute to the increments of fecal biotin levels.

Evaluation of bacterial gene expression during plant polysaccharide metabolism

A previous study indicated that starch and sucrose metabolism as plant polysaccharide metabolism was up-regulated by supplementation of fermented milk strains containing bifidobacteria[2]. Our metatranscriptome analysis showed variations in bacterial gene expression related to starch and sucrose metabolism

between the HGM and BB536-HGM groups; however, the distribution of these genes among the metabolic pathways was not consistent (Supplementary Table S7-8).

REFERENCES

1. Ifuku, O. *et al.* Origin of carbon atoms of biotin. 13C-NMR studies on biotin biosynthesis in *Escherichia coli*. *Eur. J. Biochem.* **220,** 585–91 (1994).

2. McNulty, N. P. *et al.* The impact of a consortium of fermented milk strains on the gut microbiome of gnotobiotic mice and monozygotic twins. *Sci. Transl. Med.* **3,** 106ra106 (2011).

1. Supplementary figures

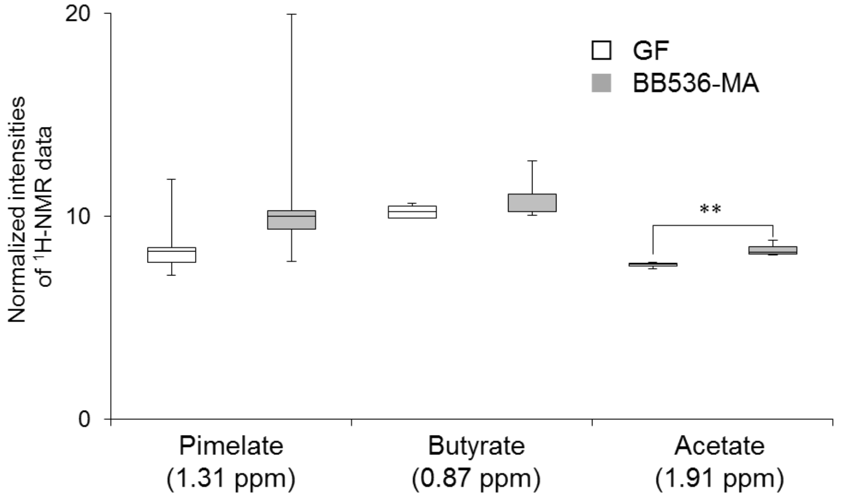

Supplementary Figure S1**.** Predicted fecal levels of pimelate, butyrate and acetate according to ^{1}H-NMR measurement.

The levels were determined according to the normalized intensities calculated from the ^{1}H-NMR measurements (n=5). Boxes denote the interquartile range between the first and third quartiles, and the lines within the boxes denote the median values. P-values were calculated using the Mann–Whitney U test. **P < 0.01.

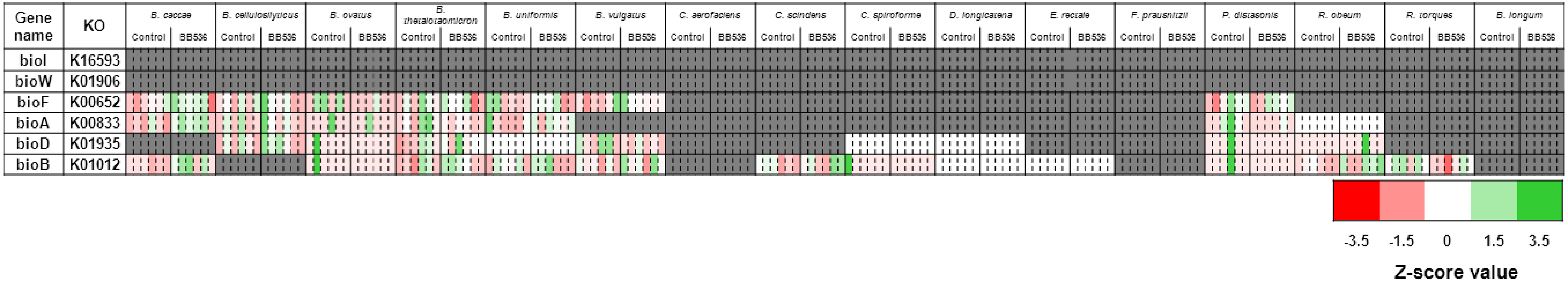

Supplementary Figure S2**.** Z-score analysis of bacterial RPKM value related to biotin synthesis.

RPKM values were used in Z-score analysis and defined by different colors, as indicated at the bottom. Each cell in the lattice represents the value of each sample. Gray color indicates that there are no genes in the database that were used in this analysis.

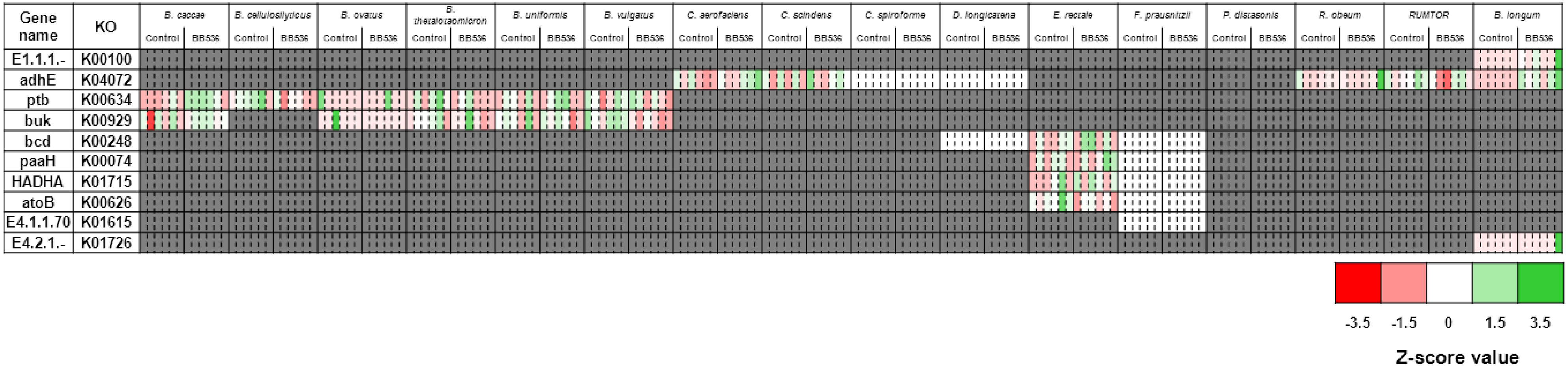

Supplementary Figure S3**.** Z-score analysis of bacterial RPKM value related to butyrate synthesis.

RPKM values were used in Z-score analysis and defined by different colors, as indicated at the bottom. Each cell in the lattice represents the value of each sample. Gray color indicates that there are no genes in the database that were used in this analysis.

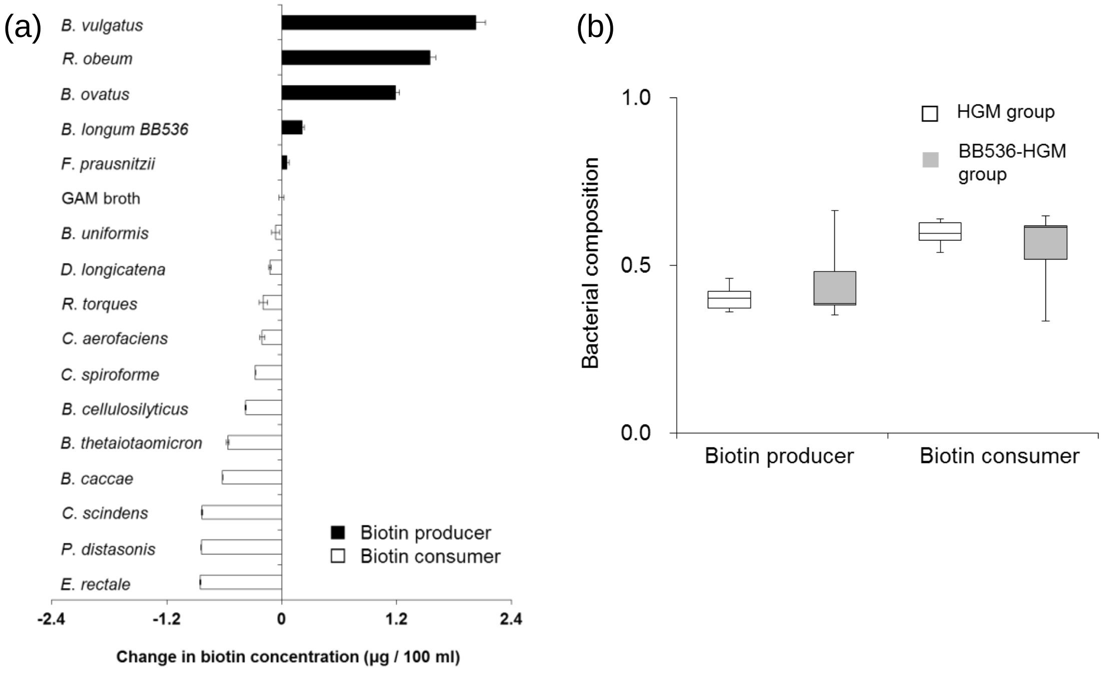

Supplementary Figure S4. Evaluation of bacterial composition according to the bacterial properties during biotin metabolism.

(a) Bacterial properties during biotin metabolism by *in vitro* assay.

Data are shown as the mean ± SD (n=3).

(b) Comparison of biotin-producing or biotin-consuming bacteria compositions between the HGM and BB536-HGM groups at day13.

Boxes denote the interquartile range between the first and third quartiles, and the lines within the boxes denote the medians.

2. Supplementary Tables

Supplementary Table S1. Bacterial gene expression of cytochrome $P450_{Biol}$ predicted by the blastX program.

Origin	Gene name	Median RPKM (interquartile range)				E-value (blastX)	P-value
		HGM group		BB536-HGM			
F. prausnitzii	FAEPRAA2165_02218	0	(0-0)	0	(0-0)	1.6E-06	NA
D. longicatena	DORLON_01762	0	(0-0)	0	(0-0)	2.8E-06	NA
B. uniformis	BACUNI_02910	1.22	(0-4.07)	0	(0-3.97)	8.7E-06	1

P-values were calculated using the Mann–Whitney U test (n=6).

Supplementary Table S2. General features of bacterial strains used for the animal study.

**Species**	**Strain**	**Taxonomy ID (NCBI)**	**Genome status**	**GenBank accession of protein-coding sequences**	**Assignment of coding sequences (KEGG Orthology)**
Bacteroides caccae	JCM9498	411901	Draft	NZ_AAVM02000001.1 - NZ_AAVM02000021.1	IMG database (version 3.5)
Bacteroides ovatus	JCM5824	411476	Draft	NZ_DS264553.1 - NZ_DS264579.1	IMG database (version 3.5)
Bacteroides thetaiotaomicron	JCM5827	226186	Complete	NC_004663.1, NC_004703.1	IMG database (version 3.5)
Bacteroides uniformis	JCM5828	411479	Draft	NZ_DS362217.1 - NZ_DS362243.1	IMG database (version 3.5)
Bacteroides vulgatus	JCM5826	435590	Complete	NC_009614.1	IMG database (version 3.5)
Bacteroides cellulosilyticus	JCM15632	537012	Draft	NZ_EQ973486.1 - NZ_EQ973551.1	IMG database (version 3.5)
Clostridium scindens	JCM6567	411468	Draft	NZ_DS499678.1 - NZ_DS499718.1	IMG database (version 3.5)
Clostridium spiroforme	JCM1432	428126	Draft	NZ_DS562843.1 - NZ_DS562854.1	IMG database (version 3.5)
Collinsella aerofaciens	JCM10188	411903	Draft	NZ_AAVN02000001.1 - NZ_AAVN02000022.1	IMG database (version 3.5)
Dorea longicatena	DSM 13814	411462	Draft	NZ_DS264384.1 - NZ_DS264411.1	IMG database (version 3.5)
Eubacterium rectale	ATCC 33656	515619	Complete	NC_012781.1	IMG database (version 3.5)
Faecalibacterium prausnitzii	DSM17677	411483	Draft	NZ_GG697149.2 - NZ_GG697168.2	IMG database (version 3.5)
Parabacteroides distasonis	JCM5825	435591	Complete	NC_009615.1	IMG database (version 3.5)
Ruminococcus obeum	ATCC 29174	411459	Draft	NZ_DS264289.1 - NZ_DS264342.1	IMG database (version 3.5)
Ruminococcus torques	ATCC 27756	411460	Draft	NZ_DS264343.1 - NZ_DS264383.1	IMG database (version 3.5)
Bifidobacterium longum	BB536	-	Complete	-	In house database

Supplementary Table S3. Constitution of modified EG medium.

Component	Amount / L
Lab-Lemco powder (Oxoid)	2.4 g
Protease peptone No. 3 (BD)	10 g
Yeast extract (BD)	5 g
Na_2HPO_4	4 g
Glucose	1.5 g
Soluble starch	0.5 g
L-cystine	0.2 g
L-cystein・HCl・H_2O	0.5 g

The pH was adjusted to 7.0. After autoclaving at 115 °C for 20 min, the medium was stocked anaerobically in glass tubes saturated with CO_2 gas.

Supplementary Table S4. Second primer list for 16S rRNA gene sequencing.

Forward primer	
Primer name	sequence (5' to 3')
F2nd_D526	AATGATACGGCGACCACCGAGATCTACAC GAGATTCC ACACTCTTTCCCTACACGACGCTCTTCCGATCTCTG
F2nd_D527	AATGATACGGCGACCACCGAGATCTACAC TTCTGAAT ACACTCTTTCCCTACACGACGCTCTTCCGATCTCTG
Reverse primer	
Primer name	sequence (5'-3')
R2nd_D701	CAAGCAGAAGACGGCATACGAGAT ATTACTCG GTGACTGGAGTTCAGACGTGTGCTCTTCCGATCTGAC
R2nd_D702	CAAGCAGAAGACGGCATACGAGAT TCCGGAGA GTGACTGGAGTTCAGACGTGTGCTCTTCCGATCTGAC
R2nd_D703	CAAGCAGAAGACGGCATACGAGAT CGCTCATT GTGACTGGAGTTCAGACGTGTGCTCTTCCGATCTGAC
R2nd_D704	CAAGCAGAAGACGGCATACGAGAT GAGATTCC GTGACTGGAGTTCAGACGTGTGCTCTTCCGATCTGAC
R2nd_D705	CAAGCAGAAGACGGCATACGAGAT ATTCAGAA GTGACTGGAGTTCAGACGTGTGCTCTTCCGATCTGAC
R2nd_D706	CAAGCAGAAGACGGCATACGAGAT GAATTCGT GTGACTGGAGTTCAGACGTGTGCTCTTCCGATCTGAC
R2nd_D707	CAAGCAGAAGACGGCATACGAGAT CTGAAGCT GTGACTGGAGTTCAGACGTGTGCTCTTCCGATCTGAC
R2nd_D708	CAAGCAGAAGACGGCATACGAGAT TAATGCGC GTGACTGGAGTTCAGACGTGTGCTCTTCCGATCTGAC
R2nd_D709	CAAGCAGAAGACGGCATACGAGAT CGGCTATG GTGACTGGAGTTCAGACGTGTGCTCTTCCGATCTGAC
R2nd_D710	CAAGCAGAAGACGGCATACGAGAT TCCGCGAA GTGACTGGAGTTCAGACGTGTGCTCTTCCGATCTGAC
R2nd_D711	CAAGCAGAAGACGGCATACGAGAT TCTCGCGC GTGACTGGAGTTCAGACGTGTGCTCTTCCGATCTGAC
R2nd_D712	CAAGCAGAAGACGGCATACGAGAT AGCGATAG GTGACTGGAGTTCAGACGTGTGCTCTTCCGATCTGAC

Supplementary Table S5. Number of sequenced reads for the 16S metagenomic analysis.

Sampling period	Group	Deposit ID		Forward primer	Reverse primer	Read for analysis
		Read1	Read2			
Day 0	HGM	PredominantCon1Day0_S636_L001_R1_00	PredominantCon1Day0_S636_L001_R2_	F2nd_D52	R2nd_D70	7073
		PredominantCon2Day0_S637_L001_R1_00	PredominantCon2Day0_S637_L001_R2_	F2nd_D52	R2nd_D70	8001
		PredominantCon3Day0_S638_L001_R1_00	PredominantCon3Day0_S638_L001_R2_	F2nd_D52	R2nd_D70	6385
		PredominantCon4Day0_S639_L001_R1_00	PredominantCon4Day0_S639_L001_R2_	F2nd_D52	R2nd_D70	6655
		PredominantCon5Day0_S640_L001_R1_00	PredominantCon5Day0_S640_L001_R2_	F2nd_D52	R2nd_D70	6249
		PredominantCon6Day0_S641_L001_R1_00	PredominantCon6Day0_S641_L001_R2_	F2nd_D52	R2nd_D70	4861
	BB536-HG	PredominantBL1Day0_S648_L001_R1_001	PredominantBL1Day0_S648_L001_R2_0	F2nd_D52	R2nd_D70	9075
		PredominantBL2Day0_S649_L001_R1_001	PredominantBL2Day0_S649_L001_R2_0	F2nd_D52	R2nd_D70	5272
		PredominantBL3Day0_S650_L001_R1_001	PredominantBL3Day0_S650_L001_R2_0	F2nd_D52	R2nd_D70	5450
		PredominantBL4Day0_S651_L001_R1_001	PredominantBL4Day0_S651_L001_R2_0	F2nd_D52	R2nd_D70	5710
		PredominantBL5Day0_S652_L001_R1_001	PredominantBL5Day0_S652_L001_R2_0	F2nd_D52	R2nd_D70	6645
		PredominantBL6Day0_S653_L001_R1_001	PredominantBL6Day0_S653_L001_R2_0	F2nd_D52	R2nd_D70	6012
Day 13	HGM	PredominantCon1Day13_S642_L001_R1_0	PredominantCon1Day13_S642_L001_R2	F2nd_D52	R2nd_D70	7489
		PredominantCon2Day13_S643_L001_R1_0	PredominantCon2Day13_S643_L001_R2	F2nd_D52	R2nd_D70	5802
		PredominantCon3Day13_S644_L001_R1_0	PredominantCon3Day13_S644_L001_R2	F2nd_D52	R2nd_D70	6595
		PredominantCon4Day13_S645_L001_R1_0	PredominantCon4Day13_S645_L001_R2	F2nd_D52	R2nd_D71	8318
		PredominantCon5Day13_S646_L001_R1_0	PredominantCon5Day13_S646_L001_R2	F2nd_D52	R2nd_D71	5450
		PredominantCon6Day13_S647_L001_R1_0	PredominantCon6Day13_S647_L001_R2	F2nd_D52	R2nd_D71	5132
	BB536-HG	PredominantBL1Day13_S654_L001_R1_00	PredominantBL1Day13_S654_L001_R2_	F2nd_D52	R2nd_D70	6074
		PredominantBL2Day13_S655_L001_R1_00	PredominantBL2Day13_S655_L001_R2_	F2nd_D52	R2nd_D70	7209
		PredominantBL3Day13_S656_L001_R1_00	PredominantBL3Day13_S656_L001_R2_	F2nd_D52	R2nd_D70	6356
		PredominantBL4Day13_S657_L001_R1_00	PredominantBL4Day13_S657_L001_R2_	F2nd_D52	R2nd_D71	6203
		PredominantBL5Day13_S658_L001_R1_00	PredominantBL5Day13_S658_L001_R2_	F2nd_D52	R2nd_D71	4402
		PredominantBL6Day13_S659_L001_R1_00	PredominantBL6Day13_S659_L001_R2_	F2nd_D52	R2nd_D71	4214

Supplementary Table S6. Number of sequenced reads for the metatranscriptomic analysis.

Sampling period	Group	Deposit ID		Barcode sequence	Mapped read
		Read1	Read2		
Day 13	HGM	9ar1_S1_L001_R2_001	9ar1_S1_L001_R2_001	ATCACG	757788
		9ar2_S2_L001_R2_001	9ar2_S2_L001_R2_001	CGATGT	877452
		9ar3_S3_L001_R2_001	9ar3_S3_L001_R2_001	TTAGGC	707926
		9ar4_S4_L001_R2_001	9ar4_S4_L001_R2_001	TGACCA	840631
		9ar5_S5_L001_R2_001	9ar5_S5_L001_R2_001	ACAGTG	837695
		9ar6_S6_L001_R2_001	9ar6_S6_L001_R2_001	GCCAAT	796974
	BB536-HGM	10ar1_S7_L001_R1_001	10ar1_S7_L001_R2_001	CAGATC	849442
		10ar2_S8_L001_R1_001	10ar2_S8_L001_R2_001	ACTTGA	831621
		10ar3_S9_L001_R1_001	10ar3_S9_L001_R2_001	GATCAG	750723
		10ar4_S10_L001_R1_001	10ar4_S10_L001_R2_001	TAGCTT	732458
		10ar5_S11_L001_R1_001	10ar5_S11_L001_R2_001	GGCTAC	613518
		10ar6_S12_L001_R1_001	10ar6_S12_L001_R2_001	CTTGTA	588301

Supplementary Table S7. Gene expression during starch and sucrose metabolism, with significant up-regulation observed in the BB536-HGM group compared with the HGM group.

Origin	Gene name	K number (KEGG)	Median RPKM (interquartile range)				P-value
			HGM group		BB536-HGM		
B. caccae	BACCAC_00071	K01187	66.4	(37.71-82.39)	155.67	(111.61-235.96)	0.041
B. caccae	BACCAC_00372	K00688	65.15	(57.71-75.19)	114.93	(100.02-134.33)	0.015
B. caccae	BACCAC_00669	K00700	39.24	(38.1-40.22)	77.94	(72.83-90.09)	0.041
B. caccae	BACCAC_00820	K01187	3.01	(2.43-3.97)	9.49	(6.34-15.58)	0.015
B. caccae	BACCAC_01310	K05349	36.61	(27.75-43.35)	71.34	(63.04-76.58)	0.004
B. caccae	BACCAC_01487	K00847	12.09	(10.02-15.08)	23.25	(20.13-23.87)	0.015
B. caccae	BACCAC_01750	K01051	1.79	(1.49-2.54)	9.31	(6.5-10.28)	0.002
B. caccae	BACCAC_03068	K00012	3.61	(2.16-4.25)	8.17	(6.52-14.33)	0.004
B. caccae	BACCAC_03604	K05349	15.88	(14.01-18.48)	24.5	(22.79-28.59)	0.009
B. cellulosilyticus	BACCELL_01545	K05349	2.43	(1.72-2.9)	4.45	(3.23-5.08)	0.041
B. cellulosilyticus	BACCELL_04938	K01187	0.26	(0-0.55)	1.01	(0.65-2.41)	0.044
B. uniformis	BACUNI_03829	K05349	0	(0-0.4)	1.42	(1.22-1.64)	0.025
C. scindens	CLOSCI_00729	K00688	31.92	(25.3-38.23)	47.18	(45.13-57.78)	0.015
C. aerofaciens	COLAER_00001	K00012	0	(0-1.36)	2.67	(1.97-5.64)	0.028
C. aerofaciens	COLAER_00012	K00703	1.39	(0.29-2.35)	4.9	(3.24-7.19)	0.013
C. aerofaciens	COLAER_01068	K00963	12.74	(10.96-13.61)	25.42	(18.06-38.63)	0.009
C. aerofaciens	COLAER_02222	K00845	6.49	(3.39-9.38)	18.55	(12.02-25.09)	0.015
R. obeum	RUMOBE_02913	K00012	0	(0-0)	1.13	(0.24-2.81)	0.028
B. longum	x0840100050	K00688	0	(0-0)	0.89	(0.17-1.38)	0.028
B. longum	x0840100463	K01835	0	(0-0)	1.46	(0.29-1.91)	0.028
B. longum	x0840100672	K01176	0	(0-0)	2.06	(1.7-2.47)	0.003

P-values were calculated using the Mann–Whitney U test (n=6).

Supplementary Table S8. Gene expression during starch and sucrose metabolism, with significant down-regulation observed in the BB536-HGM group compared with the HGM group.

Origin	Gene name	K number (KEGG)	Median RPKM (interquartile range)				P-value
			HGM group		BB536-HGM		
B. thetaiotaomicron	BT_1100	K00688	71.37	(57.2-107.75)	31.93	(27.96-42.81)	0.009
B. thetaiotaomicron	BT_2430	K00844	21.94	(16.98-24.52)	8.99	(6.62-10.52)	0.002
B. thetaiotaomicron	BT_4690	K01176	120.05	(94.31-132.54)	34.95	(26.86-42.71)	0.002
B. vulgatus	BVU_1124	K01187	31.42	(26.99-38.33)	9.48	(8.03-12.59)	0.004
B. vulgatus	BVU_1663	K01212	81.34	(65.06-96.95)	26.08	(16.66-37.08)	0.041
B. vulgatus	BVU_2776	K07405	181.37	(132.28-250.5)	90.99	(63.61-119.46)	0.041
B. vulgatus	BVU_3804	K01835	162.8	(150.43-186.94)	124.41	(98.5-150.66)	0.041
R. torques	RUMTOR_00809	K00845	67.99	(61.57-80.92)	40.04	(36.64-42.85)	0.041
R. torques	RUMTOR_00928	K00703	241.31	(190.9-323.88)	146	(120.57-169.01)	0.041
R. torques	RUMTOR_01163	K00705	53.01	(48.1-56.67)	25.46	(17.17-33.93)	0.009
R. torques	RUMTOR_01198	K00845	10.13	(6.35-13.48)	2.75	(1.53-5.2)	0.026
R. torques	RUMTOR_01250	K00975	161.59	(121.09-393.17)	99.7	(72.58-119.82)	0.041
R. torques	RUMTOR_01411	K00975	243.65	(219.92-462.78)	177.08	(139.12-208.47)	0.041
R. torques	RUMTOR_01412	K00975	294.69	(250.47-490.44)	205.69	(169.36-217.42)	0.026
R. torques	RUMTOR_01422	K00700	140.97	(137.98-222.87)	106.91	(71.26-115.86)	0.009
R. torques	RUMTOR_01462	K01835	164.53	(139.36-241.17)	108.2	(72.18-123.3)	0.041
R. torques	RUMTOR_01473	K01187	26.45	(22.09-32.06)	15.36	(11.6-21.28)	0.041
R. torques	RUMTOR_02229	K01810	198.95	(168.9-298.06)	119.94	(91.04-138.41)	0.041
R. torques	RUMTOR_02677	K00688	51.05	(46.07-62.25)	30.61	(19.43-35.83)	0.041

P-values were calculated using the Mann–Whitney U test (n=6).
